# Multidrug resistant *Proteus mirabilis* and *Escherichia coli* causing fulminant necrotising fasciitis: a case report

**DOI:** 10.1186/s13104-018-3413-7

**Published:** 2018-05-21

**Authors:** Eugene Vernyuy Yeika, Joyce Bei Foryoung, Derrick Tembi Efie, Eugene Adze Nkwetateba, Paul Nkemtendong Tolefac, Marcelin Ngowe Ngowe

**Affiliations:** 1 Saint Elizabeth Catholic General Hospital and Cardiac Centre Shisong, PO Box 8, Kumbo, Cameroon; 2 Clinical Research Education Networking and Consultancy, Douala, Cameroon; 3 Health and Human Development Research Group, Douala, Cameroon; 4 Faculty of Health Sciences, University of Buea, Buea, Cameroon

**Keywords:** Necrotizing fasciitis, Multidrug resistance, Empiric antibiotics, Case report

## Abstract

**Background:**

Necrotizing fasciitis is a rare soft tissue infection characterized by rapid progressive necrosis with relative sparing of underlying muscles. This case is reported to highlight the emergence of multidrug resistant microbes in recent days which limits the use of empiric antibiotic therapy and necessitates early cultures and sensitivity enabling targeted antibiotic therapy. Factors that lead to antimicrobial resistance especially in sub-Saharan Africa have also been discussed.

**Case presentation:**

We report the case of a 52-year-old black man who was referred to our centre for the management of cellulitis and suppurating ulcers of the right leg which had progressed to a wet gangrene. Following physical examination and work-up, a diagnosis of fulminant necrotizing fasciitis of the right leg caused by multidrug resistant *Proteus mirabilis* and *Escherichia coli* was made. Despite the broad-spectrum empiric antibiotic therapy and aggressive multiple surgical debridement, necrosis progressed leading to an above-knee amputation.

**Conclusion:**

Necrotizing fasciitis is a surgical emergency that requires prompt diagnosis and aggressive surgical debridement in order to reduce morbidity and mortality. The emergence of multidrug resistant organisms in recent days have limited the use of empiric antibiotic therapy, necessitating early culture and sensitivity and the use of susceptibility-guided antibiotic therapy. Timely action to control the use of antibiotics in sub-Saharan Africa will reduce multidrug resistance and delay the arrival of post-antibiotics era.

## Background

Necrotizing fasciitis (NF) is a rare soft tissue infection characterized by rapid progressive necrosis with relative sparing of underlying muscles [1, 2]. Patients with NF usually present with nonspecific features like fever, excruciating pain, oedematous and erythematous skin lesions that often rapidly deteriorate to haemorrhagic blebs or fluid-filled bullae and gangrenous necrosis [3, 4]. Over 70% of cases of NF are caused by polymicrobial organisms with most cultures yielding a mixture of aerobic and anaerobic organisms [5–7]. The most common pathogens isolated from cultures are gram positive organisms like *β*-hemolytic group A streptococcus, *Staphylococcus aureus*, bacillus species, enterococci species and gram negative organisms such as *Klebsiella pneumonia, Pseudomonas aeroginosa*, *Serratia species*, *Escherichia coli*, *Clostridium* species, *Fusobacterium* species, and *Prevotella* species [2, 7, 8]. NF carries a high morbidity and mortality especially when diagnosed late [5, 9] necessitating prompt diagnosis and timely treatment with radical surgical debridement and empiric broad spectrum antibiotic therapy [2, 10]. The mortality rate due to NF ranges from 25 to 35% despite empiric broad-spectrum antibiotic therapy and surgical debridement [11]. This case is reported to highlight that the emergence of multidrug resistant organisms (MDRO) limits the use of empiric antibiotic therapy and necessitates early cultures and sensitivity to enable targeted antibiotic therapy. The factors that lead to antimicrobial resistance especially in sub-Saharan Africa (SSA) had also been discussed. MDR is defined as non-susceptibility to at least one agent in three or more antimicrobial categories [12].

## Case presentation

A 52-year-old black man with no relevant past medical history was referred to our centre for the management of cellulitis. He presented with a swollen erythematous and painful right leg that progressed to formation of blebs, suppurating ulcers and a wet gangrene. This started at knee joint 8 days prior to presentation and was initially managed with over-the-counter amoxicillin and diclofenac. The swelling later progressed to involve the right leg with development of blister-like lesions 4 days following the onset of symptoms. The patient also developed generalised pruritic rash prompting consultation at the regional hospital and there, a diagnosis of a sepsis secondary to a cellulitis was made. Initial work-up at the regional hospital comprised of a complete blood count which revealed leucocytosis (white cell count of 53.9 × 10^9^/l) with neutrophil predominance, normocytic normochromic anaemia (haemoglobin of 3.9 g/dl) and thrombocytosis (platelet count of 880 × 10^9^/l). The blood urea nitrogen was 114 mg/dl and serum creatinine was 2.1 mg/dl (l84.8 µmol/l). The patient was initially managed with intravenous ceftriaxone 1 g 12 hourly, intravenous metronidazole 500 mg 8 hourly and subcutaneous enoxaparin 4000 IU 24 hourly and was also transfused two units of compatible whole blood. The patients’ stay at the regional hospital was marked by persistence of fever, progressive ulceration of the leg, bullae formation and the onset of gangrene prompting referral to Saint Elizabeth Catholic General Hospital Shisong following 4 days of hospitalisation.

**Fig. 1:**
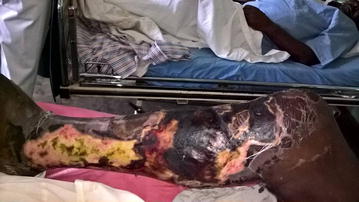
Extensive necrotizing fasciitis of the right leg

Physical examination upon arrival at our centre revealed a temperature of 38.9 °c, respiratory rate of 23 breaths/min, blood pressure of 99/70 mmHg and a pulse rate of 114 beats/min. The patient was alert with pale conjunctivae and anicteric sclerae and his weight was 66 kg. His left leg was swollen, warm, fluctuant with ulcerated skin and necrosis to the level of the knee joint (Fig. 1). All digits of the right foot were pale and cold with absent posterior tibial pulse. The following investigations were conducted: a random blood glucose was 150 mg/dl (8.3 mmol/l) and a fasting blood glucose done 16 h later was 117 mg/dl (6.5 mmol/l). A complete blood count revealed a white cell count of 16.4 × 10^9^/l with neutrophil predominance, a microcytic hypochromic anaemia with haemoglobin level of 7.2 g/dl and platelet count of 569 × 10^9^/l. A diagnosis of fulminant necrotizing fasciitis was made.

**Fig. 2:**
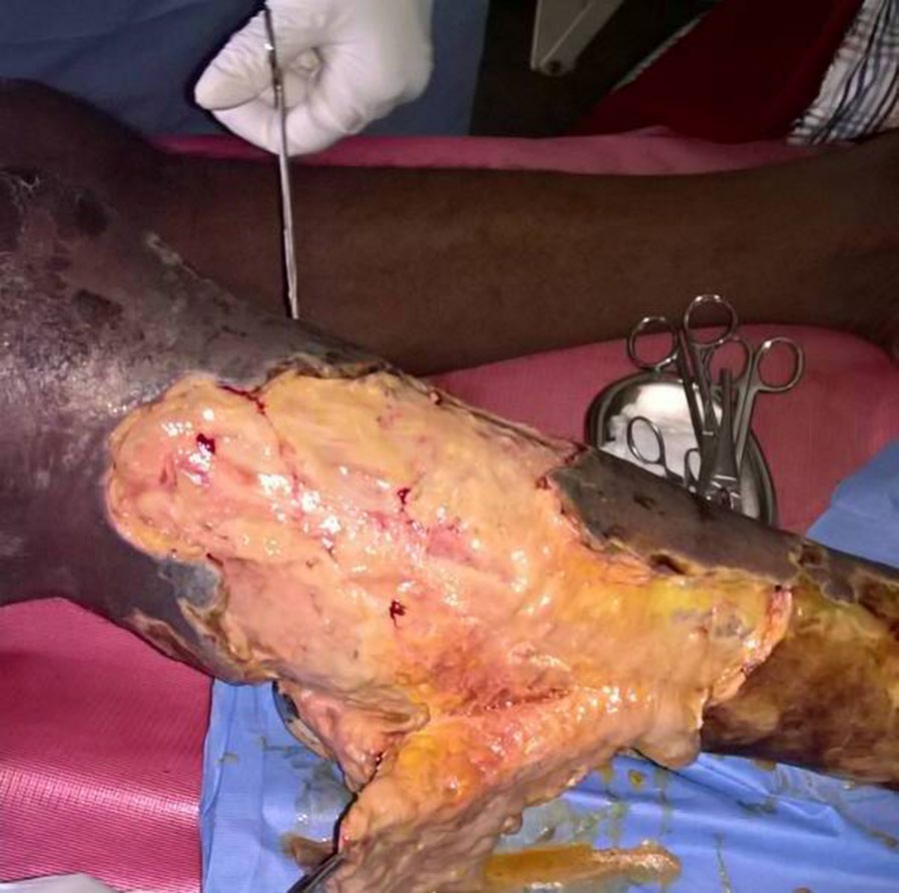
Extensive tissue necrosis during debridement

**Fig. 3:**
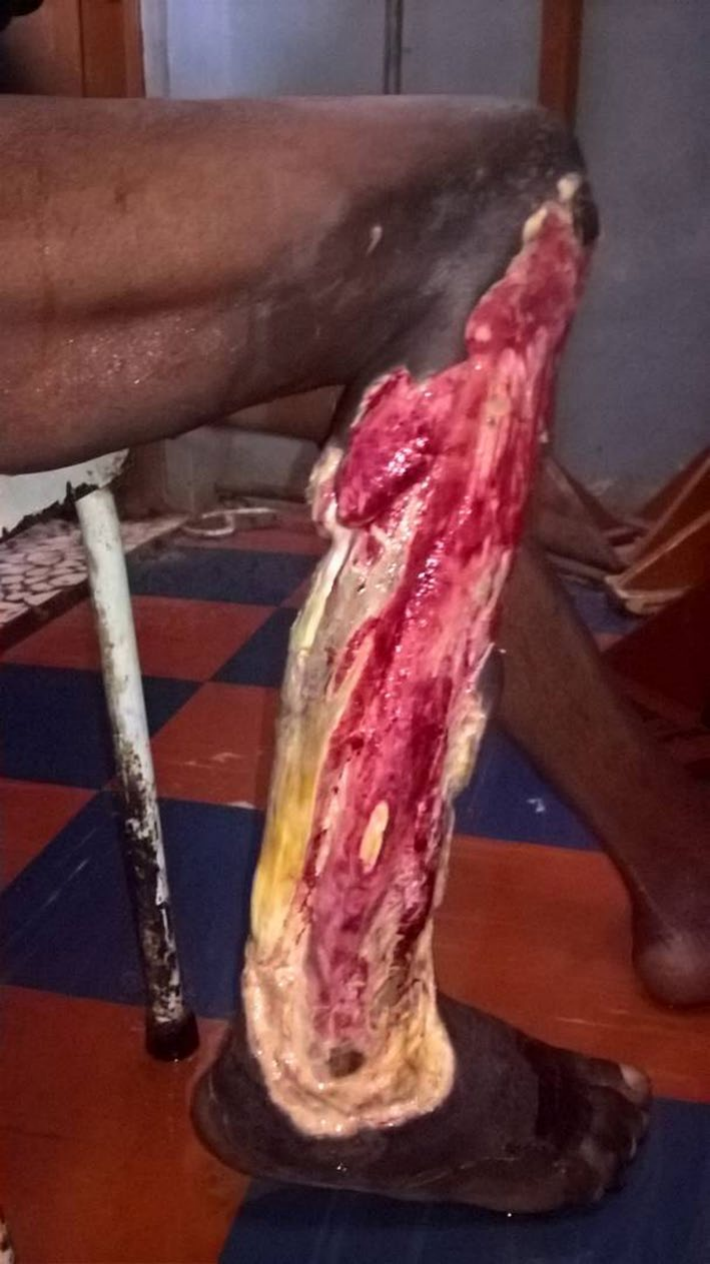
Shameful exposure of the muscles of the leg after debridement

**Fig. 4:**
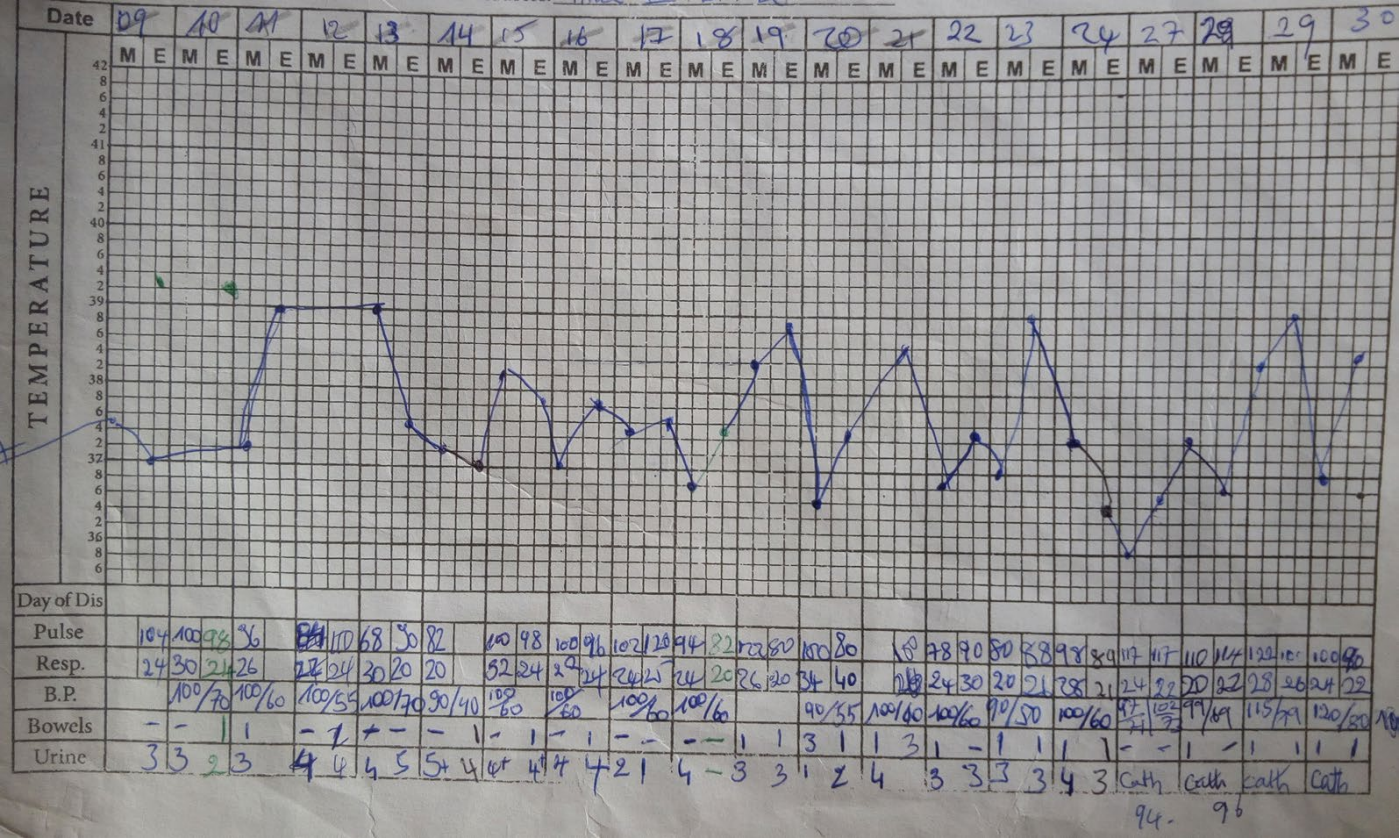
Vital signs chart showing swinging pyrexia

**Fig. 5:**
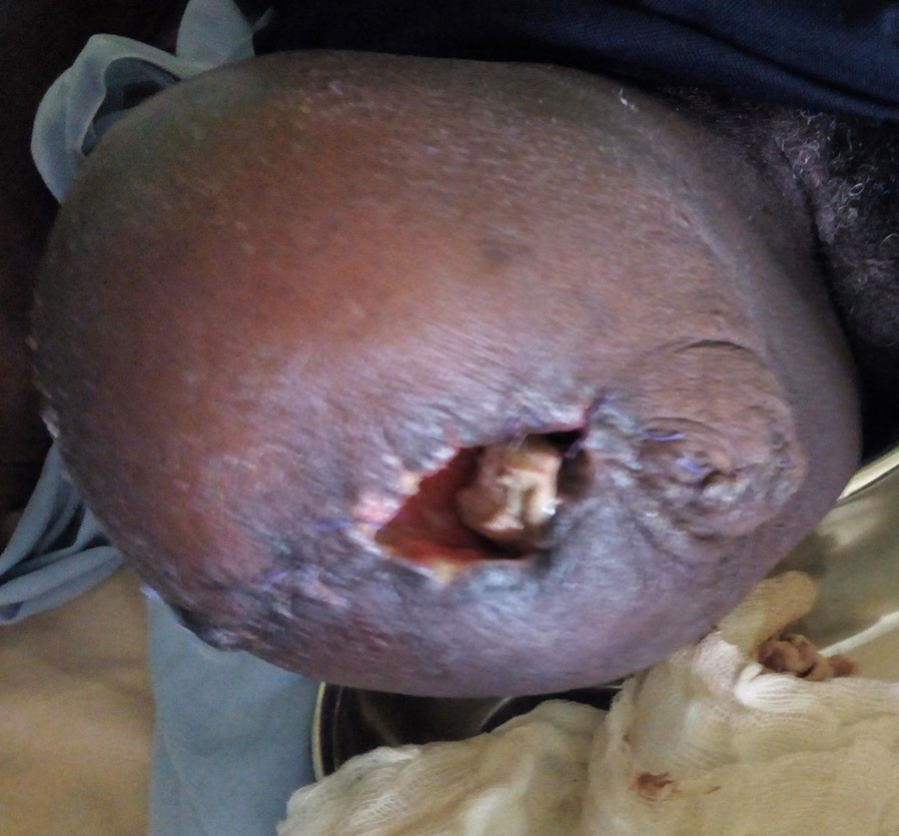
Infected amputated stump by MDR *Proteus mirabilis*

The following parenteral antibiotics were given upon admission: ampicillin 1 g 6 hourly, gentamycin 80 mg 12 hourly and metronidazole 500 mg 8 hourly; analgesia included intravenous paracetamol 1 g 6 hourly and fluids. After counselling and consent, surgical debridement was done under general anesthesia 12 h following hospitalization. Visualization during surgical exploration confirmed the diagnosis of NF (Figs. 2 and 3). Debridement was repeated on day 3 and day 7 of hospitalization. After debridement, the wound was washed and dressed 12 hourly with Dakin’s solution and normal saline. A wound swab was sent for culture and sensitivity and *Proteus mirabilis* and *E. coli* were isolated. Necrosis progressed despite multiple and aggressive surgical debridement resulting to an above-knee amputation. Two units of compatible whole blood were transfused during surgery. The patient continued having swinging pyrexia (Fig. 4) and on post-operative day 6, the stump started producing purulent discharges (Fig. 5). The second wound swab was collected and sent for culture and sensitivity and it still revealed *P. mirabilis* and *E. coli* were isolated. *Proteus mirabilis* was sensitive only to the carbapenems (imipenem and meropenem) while *E. coli* was additionally sensitive to ofloxacin and ornidazole (Table 1).

**Table 1:** Antibiogram results showing MDR

	Antibiotics	Sensitivity to *Proteus mirabilis*	Sensitivity to *Escherichia coli*
1	Amoxicillin	R	R
2	Amoxicillin + clavulanic acid	R	R
3	Cloxacillin	R	R
4	Ceftriaxone	R	R
5	Cefixime	R	R
6	Erythromycin	R	R
7	Clarithromycin	R	R
8	Azithromycin	R	R
9	Gentamycin	R	R
10	Doxycycline	R	R
11	Clindamycin	R	R
12	Chloramphenical	R	R
13	Thiamphenicol	R	R
14	Ciprofloxacin	R	R
15	Gatifloxacin	R	R
16	Laevofloxacin	R	R
17	Metronidazole	R	R
18	Ciprofloxacin + tinidazole	R	R
19	Ofloxacin + ornidazole	R	S
20	Nitrofurantoin	R	R
21	Imipenem	S	S
22	Meropenem	S	S

*R* resistant, *S* sensitive

Further management involved removal of sutures, debridement of necrosed tissue and initiation of parenteral meropenem 1 g given 8 hourly. Clinical progress was marked by formation of granulation tissue. The wound was closed up 6 days later and the patient was eventually discharged after 7 weeks of hospitalization.

## Discussion and conclusion

Multidrug resistance have been frequently reported in recent days and threatens the effectiveness of successful treatment of infections especially using empiric antibiotics. The incidence of MDR microbes is on the rise over the past decades, meanwhile many studies still advocate for early broad-spectrum empiric or combination antibiotic therapy [9]. Godebo et al. in a study to determine multidrug resistance rate of bacterial isolates that caused wound infections in a specialised centre in SSA, showed that overall MDR among gram positive and gram negative bacterial isolates were 77 and 59.3% respectively [13]. The selection of appropriate antimicrobial agents for any suspected NF must take into account the nature of patient’s exposure and local epidemiologic data [9]. Empiric antibiotic therapy is limited because it cannot be used in the context of MDRO. This is the situation in this case report as culture and sensitivity results revealed resistance to all the antibiotics previously used. Some pathogens also possess the ability to develop new or ongoing resistance during treatment [9], further complicating the blind use of antibiotics. Progressive necrosis of soft tissues despite the empiric use of antibiotic therapy is a big indicator of MDR and warrants early culture and sensitivity to enable the use of susceptibility-guided antibiotic therapy. Routine and early culture and sensitivity is a means for early detection of MDRO and early use of susceptibility-guided antibiotic therapy should be done at the level of referring hospitals. This does not only reduce morbidity and mortality, but also reduces the length of hospital stay and the cost of hospitalisation.

The key to successful management of patients with necrotizing soft tissue infections relies on early recognition, prompt and aggressive surgical debridement with targeted antibiotic therapy [3, 10, 14]. Early diagnosis of NF remains a challenge partly due to nonspecific skin findings causing it to be misdiagnosed as cellulitis [2, 5, 14]. This patient was initially managed as cellulitis prior to referral, and this delayed the diagnosis of NF. Such delays in recognition and treatment will result in greater soft tissue lost and increased risk of morbidity and mortality [5, 14]. Early clinical differentiation between NF and cellulitis is important for early surgical management. Kobayashi et al. showed that delays in surgical treatment of > 12 h are associated with an increased number of surgical debridement, higher incidence of septic shock and acute kidney injuries in patients with necrotizing soft tissue infections [15]. Unusual location of soft tissue infections, lack of associated co-morbidities and/or risks factors, absence of any history of preceding trauma or an obvious breech in the continuity of skin or mucosa, or a low Laboratory Risk Indicator for Necrotizing Fasciitis (LRINEC) score thus excludes the diagnosis of NF [2, 4]. Our patient presented no specific risk factors for NF and no history of initiating trauma or breech in skin continuity but however developed a life threating NF.

Although NF is associated with a high morbidity and mortality, early diagnosis and surgical debridement have shown a favourable outcome making it not just a medical but also a surgical emergency [5]. The decision for surgical debridement often comes late due to late diagnosis. The LRINEC scoring system is used to assist in early diagnosis of NF [5]. This is the only validated diagnostic tool for NF currently in use and carries a positive predictive value of 92% [16]. This tool is based on six parameters at the time of presentation; C-reactive protein, total white cell count, haemoglobin, serum sodium, creatinine and glucose. A LRINEC score of 6 or more confers a higher risk of NF [16]. The LRINEC scoring system has not yet achieved wide-spread use due to some investigations like C-reactive protein which requires over 24 h for the results and is absent in most resource-limited settings. The controversial views of some authors with many papers questioning its usefulness in early recognition of LRENIC in recognising NF have also limited it use [2, 17]. Many studies have validated the ability of the LRINEC in detecting NF and differentiating it from other soft tissue infections like cellulitis that may clinically present in a similar fashion while others haven’t [16, 18]. The LRINEC score is not adequately sensitive despite its high specificity, and consequently a low LRINEC score cannot be used to eliminate the diagnosis of NF [2, 19]. According to Patel and associates, the diagnosis of NF still heavily relies on clinical findings such as pain, fever and hemodynamic instability [2]. In our patient, we gathered a LRINEC score of 6 suggestive of NF despite the unavailability of C-reactive protein, warranting a surgical exploration. Modifying the LRINEC scoring system to include both clinical and laboratory findings is therefore necessary to improve the specificity and sensitivity of this scoring system and make it more useful in resource limited settings.

Multidrug resistance has become a public health issue with national and global dimensions. There are many factors that contribute to the development of antibiotic resistance including the absence of quality assurance and antibiotic surveillance in most parts of SSA. Treatment with sensitive antibiotics is not always evident in resource-limited settings due to cost and unavailability of most antibiotics. The existence of very few centres that can conduct cultures and sensitivity in most countries in SSA have prompted the inevitable use of empiric or combined antibiotics. Due to the poor socioeconomic status of patients, expensive antibiotics are avoided for empiric treatment and can only be prescribed only following antimicrobial culture and sensitivity, however very few patients also afford for culture and sensitivity. Due to lack of government policies restricting over-the-counter sales of drugs especially antibiotics, many patients have resorted to the use of self-prescribed antibiotics prior to consultation which lead to usage of poor quality of drugs, sub-therapeutic doses and non-respect of therapeutic durations.

In recent years where MDR is frequently reported in many parts of the world, we recommend the adoption and use of Center for Infectious Disease Control guidelines for management of MDRO. According to these guidelines, the following strategies are inevitable means to prevent, curb or reduce MDR: administrative support in terms of government policies limiting the sales of over-the-counter antibiotics, creation of antibiotics surveillance departments in the public health ministries and education of the masses on the judicious use of antimicrobial agents. The diversity of potential pathogens resistant to commonly prescribed antibiotics underscores the importance of sustained and standardized antimicrobial resistance surveillance and antibiotic stewardship programmes in developing countries [20], yet these programs are grossly absent in SSA.

## Abbreviations

LRINEC: Laboratory Risk Indicator for Necrotizing Fasciitis
MDR: multidrug resistance
MDRO: multidrug resistant organisms
NF: necrotizing fasciitis
SSA: sub-Saharan Africa
